# Irradiation differentially affects substratum-dependent survival, adhesion, and invasion of glioblastoma cell lines

**DOI:** 10.1038/sj.bjc.6601429

**Published:** 2003-11-25

**Authors:** N Cordes, B Hansmeier, C Beinke, V Meineke, D van Beuningen

**Affiliations:** 1 Institute of Radiobiology, German Armed Forces, Neuherbergstrasse 11, 80937 Munich, Germany

**Keywords:** *β*1-integrin, *β*3-integrin, matrix metalloproteinase-2, invasion, glioblastoma

## Abstract

Effects of ionising radiation on extracellular matrix (ECM)-modulated cell survival and on adhesion and invasion are not well understood. In particular, the aggressiveness of glioblastoma multiforme has been associated with tumour cell invasion into adjacent normal brain tissue. To examine these effects in more depth, four human glioblastoma cell lines (A-172, U-138, LN-229 and LN-18) were irradiated on fibronectin (FN), Matrigel, BSA or polystyrene. Major findings of this study include a significantly increased survival of irradiated A-172 but not of irradiated U-138, LN-229, and LN-18 cells on FN or Matrigel compared to cells irradiated on polystyrene or BSA. Irradiation induced a dose-dependent increase in functional *β*1- and *β*3-integrins in all four glioma cell lines. This integrin induction caused improved cell adhesion to FN or Matrigel. In contrast to U-138, LN-229 and LN-18 cells, irradiation strongly impaired A-172 cell invasion. Invasion of all cell lines was inhibited by anti-integrin antibodies, the disintegrin echistatin and the MMP-2/-9 inhibitor III. Additionally, *β*1- and *β*3-integrins modulated basal and radiation-altered gelatinolytic activity of MMP-2. Tested glioblastoma cell lines showed a differential cellular susceptibility to FN or Matrigel which affected the cellular radiosensitivity. Three out of four glioma cell lines demonstrated a combination of a substratum-independent survival after irradiation and an invasive potential which was not affected by irradiation. *β*1- and *β*3-integrins were identified to play a substantial, regulatory role in survival, adhesion, invasion and MMP-2 activity. Detailed insights into radioresistance and invasion processes might offer new therapeutic strategies to enhance cell killing of lethal high-grade astrocytoma.

High-grade astrocytomas such as glioblastoma multiforme (GBMs) are the most common primary brain tumours in adults (Burger *et al*, 1985). In addition to surgical resection, the standard clinical management of this tumour entity involves radiotherapy with or without adjuvant chemotherapy (Kristiansen *et al*, 1981; Fisher *et al*, 2001; Stupp *et al*, 2002). In spite of this multidisciplinary approach, the prognosis for patients with GBM remains poor (Levin *et al*, 1997; Galanis and Buckner, 2000).

GBMs are characterised by massive and diffuse infiltration of the surrounding normal brain tissue and they rarely metastasise to peripheral organs (Kleihues *et al*, 1993). These findings suggest distinctive mechanisms but not distinctive key molecules that regulate cell invasion of brain tumour cells. Important key molecules that enable cell invasion are extracellular matrix (ECM)-degrading enzymes (Liotta and Stetler-Stevenson, 1990; Stetler-Stevenson *et al*, 1993) like matrix metalloproteinases (MMPs) (Ennis and Matrisian, 1994), cathepsin family proteases (Rochefort, 1994) and plasminogen activators (Mignatti and Rifkin, 1996). To date, 26 different MMPs are known and GBMs frequently show an elevation of various MMPs (Gokaslan *et al*, 1998; Nakano *et al*, 1995). In particular, MMP-2 (Brooks *et al*, 1996) and MMP-9 (Rao *et al*, 1993) render critical for glioma cell invasion. Additionally, irradiation was found to induce the catalytic activity, mRNA and protein synthesis of MMP-2 (Engebraaten *et al*, 1992; Bauman *et al*, 1999; Wild-Bode *et al*, 2001). The clinical consequences and interpretation of these findings for glioma cell migration and invasion remain controversial.

To invade a cell requires both specific ECM-degrading proteinases and specific cell surface receptors of the integrin family (Albelda, 1993; Hynes, 2002). Integrins comprise a large group of at least 24 different heterodimeric transmembrane receptors that govern cell–ECM interactions (Hynes, 2002). Each heterodimer consists of one *α*- and one *β*-chain. Integrins are involved in the regulation of several cell functions such as differentiation, cell cycle progression, apoptotic pathways and DNA repair mechanisms (Adams and Watt, 1990; Assoian, 1997; Hazlehurst *et al*, 2001). To date *α*v*β*3 (Brooks *et al*, 1996; Rooprai *et al*, 1999), *α*5*β*1 (Ohnishi *et al*, 1998) and other *β*1-integrin combinations (Paulus *et al*, 1993; Chintala *et al*, 1996; Friedlander *et al*, 1996) have been identified to participate in cell migration and invasion. The role of the *α*v*β*3-integrin for migration has been examined in detail and evidence was provided that this integrin has a dual function. It is a binding receptor for ECM proteins and it serves as a receptor for MMP-2 on the cell surface (Brooks *et al*, 1996). These interactions facilitate conversion of pro-MMP-2 to active MMP-2 thereby controlling both migration and proteolytic cleavage of ECM components. Similarly, *β*1-integrins have also been uncovered to control the expression and activity of MMP-2 (Ellerbroek *et al*, 1999) and MMP-9 (Troussard *et al*, 2000).

In addition to cell invasion, interactions between cells and the ECM regulate survival and proliferation (Meredith *et al*, 1993; Schwartz, 2001). Previously, we showed an ECM-dependent reduction of the cellular radiosensitivity, termed cell adhesion-mediated radioresistance (CAM-RR), in a variety of malign and normal cell lines (Cordes and Meineke, 2003; Cordes and van Beuningen, 2003). We also reported a radiation-induced upregulation of functional *β*1-integrins in human lung cancer cell lines that strongly improved adhesion to fibronectin (FN) or laminin (Cordes *et al*, 2002, in press (a), (2003)).

To further define the interactions of irradiation and *β*1-, *β*3-integrin and MMP-2, ECM-mediated cell survival and *β*1- and *β*3-integrin-dependent cell adhesion and invasion was examined in four human glioma cell lines with different invasive and tumourigenic potentials.

## MATERIALS AND METHODS

### Cells

A-172 cells were deposited from DJ Giard and U-138 cells were from the German Cancer Research Centre in Heidelberg. Both cell lines were found to form colonies in semisolid medium but were not tumorigenic in immunosuppressed mice. LN-229 and LN-18 cells were established from de Tribolet and both cell lines were found to form tumours in nude mice. All glioblastoma cell lines used were purchased from the American Type Culture Collection (ATCC, Rockville, MD, USA). Dulbecco's modified Eagle's medium (DMEM; GIBCO, Germany) supplemented with 10% foetal bovine serum (FBS; PAA, Austria) and 1% nonessential amino acids (GIBCO, Germany) was applied to culture the cells routinely at 37°C in a humidified atmosphere containing 10% CO_2_ buffered at pH 7.35. Where indicated, serum starvation of cells was performed using DMEM supplemented with 1% nonessential amino acids without FBS. For all experiments asynchronous, exponentially growing cell cultures were employed.

### Radiation exposure

Irradiation was delivered at room temperature using single doses of 240 kV X-rays (Isovolt 320/10; Seifert, Germany) filtered with 3 mm Be. The absorbed dose was measured using a Duplex dosimeter (PTW, Germany). The dose rate was approximately 1 Gy min^−1^ at 13 mA. Applied doses ranged from 0 to 8 Gy.

### siRNA transfection

The sequence of *β*1- and *β*3-integrin siRNA was selected based on a method described previously (Elbashir *et al*, 2002). The target sequence that effectively mediates silencing of *β*1-integrin expression is 5′-AATGTAACCAACCGTAGCA-3′ and of *β*3-integrin expression is 5′-CAAGCCTGTGTCACCATAC-3′ (sense sequences). The 21-nucleotide synthetic siRNA duplex was prepared by MWG (Germany) based on Dharmacon 2′-ACE technology. A-172 and U-138 cells were transfected with the *β*1- or *β*3-integrin siRNA or a 21-nucleotide irrelevant RNA Duplex II as a control using oligofectamine (Invitrogen, Germany). Depletion of *β*1- and *β*3-integrin was confirmed by Western blotting.

### Colony formation assay

The colony formation assay was applied for measurement of clonogenic cell survival. Cells were plated onto polystyrene (Falcon, Germany) or FN (Becton Dickinson, Germany), Matrigel®-Matrix (Becton Dickinson, Germany) or bovine serum albumin (BSA; Sigma-Aldrich, Germany) precoated six-well dishes (Falcon, Germany). Culture plates and flasks were coated with 1 *μ*g cm^−2^ FN or BSA or 100 mg ml^−1^ Matrigel in serum-free medium for 1 h at room temperature. SiRNA-transfected A-172 and U-138 cells were seeded on polystyrene and irradiated with 2 or 6 Gy. Cells were exposed to radiation 24 h after plating. At 8–10 days after irradiation, grown colonies were stained with Coomassie blue. Colonies greater 50 cells were counted. All experiments were repeated three times.

### Flow cytometry

Determination of *β*1- and *β*3-integrin cell surface expression was performed as published elsewhere (Cordes *et al*, 2002). Shortly, subconfluent monolayer cell cultures (∼60% confluence) attached to polystyrene, FN or Matrigel were irradiated with 2 or 6 Gy, detached with trypsin/EDTA at defined time points (0, 12, 24, 36, 48 h) and prepared for measurements. Nonirradiated controls were prepared in parallel at each time point. Staining with FITC-conjugated anti-*β*1-integrin IgG antibodies (Dako, Germany) or anti-*β*3-integrin antibodies (Pharmingen, Germany) was achieved for 1 h at room temperature. Isotype IgG control was from Serotec (Germany) and secondary FITC-conjugated anti-mouse antibodies were from Dianova (Germany). Finally, prepared cells were measured using a Fluorescence-Activated Cell Sorter (FACSCalibur; Becton Dickinson, Germany) equipped with the CELLQuest software. Experiments were repeated three times.

### Adhesion assay

The cell adhesion to FN, Matrigel, BSA or polystyrene was studied according to a method described previously (Cordes *et al*, 2002). In brief, 96-well plates (Nunc, Germany) were coated with 1 *μ*g cm^−2^ FN or BSA or 100 mg ml^−1^ Matrigel in serum-free medium for 1 h at room temperature. At 48 h postirradiation, 5x10^4^ nonirradiated, irradiated (6 Gy) or siRNA-transfected cells grown on polystyrene were washed, trypsinised and plated onto prepared wells in the absence or presence of adhesion-blocking *β*1-integrin antibodies (mAb13, 10 *μ*g ml^−1^, Pharmingen, Germany), adhesion-blocking *β*3-integrin antibodies (RUU-PL7F12, 10 *μ*g ml^−1^, Pharmingen, Germany) or the *β*1/*β*3-integrin-blocking disintegrin echistatin (10 *μ*M, Sigma-Aldrich, Germany) (Staiano *et al*, 1995). Unspecific IgG2a (Upstate, Germany) and IgG1 (Pharmingen, Germany) antibodies were employed for control experiments using identical concentrations as done for specific antibodies. After 45 min incubation at 37°C, nonadherent cells were withdrawn by gentle washing with PBS. Adherent cells were fixed with 70% ethanol and stained using 1% methylene blue. Washing with distilled water was followed by the addition of 100 μl of an 0.1 M HCl solution to each well and measurement of absorbance of the resulting solution at 630 nm using a spectral-photometer (Spectra max® 190, molecular devices, Germany). All experiments were performed in triplicate and repeated three times.

### *In vitro* invasion assay

The potential of invasion of nonirradiated and irradiated (6 Gy) as well as siRNA-transfected cells into Matrigel was determined by the use of polycarbonate filters (8-*μ*m pore size; Becton Dickinson, Germany) coated with growth factor-reduced Matrigel placed in a modified Boyden chamber. Cells (2.5x10^4^) were added to the top chamber. Top and bottom chambers contained media with 10% FBS. This experimental set-up was chosen according to the flow cytometric analysis of *β*1- and *β*3-integrins to enable direct evaluation of the role of *β*1- and *β*3-integrins for cell invasion. After a 24-h time interval, the FBS-containing medium was changed to serum-free medium shortly prior to irradiation with 6 Gy. At 48 h post radiation, which presented the time point of *β*1- and *β*3-integrin upregulation, cells in the top chamber were incubated with 25 *μ*M MMP-2/-9 inhibitor III (Calbiochem-Novabiochem, Germany) (Koivunen *et al*, 1999), 10 *μ*g ml^−1^ anti-*β*1-integrin antibodies mAb13, 10 *μ*g/ml^−1^ anti-*β*3-integrin antibodies RUU-PL7F12 or 10 μM *β*1/*β*3-integrin-blocking echistatin for 15 min. Unspecific IgG2a and IgG1 antibodies were employed for control experiments using identical concentrations as done for specific antibodies. Replacing serum-free medium in the lower chamber with 10% FBS-containing medium started the assay. After 24 h, noninvaded cells were wiped off the top surface of the membrane filter with a cotton swap and filters were removed. Invaded cells were fixed with 70% ethanol and 4% paraformaldehyde, stained with Coomassie blue and enumerated in four high-powered fields using a Leitz Diaplan microscope (Bensheim, Germany; × 10 magnification). Cells were counted twice by two independent observers (NC and BH). Interobserver variation was < 5%.

### Total protein extracts and Western blotting

Nonirradiated or irradiated cells (6 Gy) were harvested with trypsin/EDTA 48 h postirradiation. Subsequent to resuspension of cells in ice-cold medium, centrifugation at 4°C and washing with ice-cold PBS, cells were lysed using modified RIPA buffer containing 50 mM Tris-HCl (pH 7.4), 1% NP-40, 0.25% sodium deoxycholate, 150 mM NaCl, 1 mM EDTA supplemented with protease inhibitor cocktail complete® (Roche, Germany), 5 mM sodium vanadate and 5 mM sodium fluoride. Cell homogenisation was performed with a 25-gauge needle. Total protein extracts were stored at –134°C until use. SiRNA-transfected cells were lysed with RIPA buffer 48 h after transfection. Total protein extracts (20 *μ*g) were separated by SDS – polyacrylamide electrophoresis on 10% (MMP-2, MT1-MMP, *β*1-integrin, *β*3-integrin) or 15% (TIMP-2) gels. Transfer of separated proteins onto a nitrocellulose membrane (Schleicher & Schuell, Germany), blocking membranes with 5% nonfat dry milk powder in PBS and incubation of primary antibodies at 4°C overnight were followed. The following antibodies were used: anti-MMP-2 (1 : 500), anti-MT1-MMP (1 : 500), anti-TIMP-2 (1 : 40) (Oncogene, Germany), anti-*β*1-integrin (1 : 500), anti-*β*3-integrin (1 : 500) (Transduction Laboratories, Germany) and anti-*β*-actin (1 : 2000) (Sigma-Aldrich, Germany). The protein detection was accomplished using specific horseradish peroxidase-conjugated goat antimouse antibodies (Santa Cruz, Germany) in combination with the enhanced chemiluminescence detection system (ECL, Amersham, Life Sciences, Germany).

### Immunofluorescence staining and laser confocal scanning microscopy

Immunofluorescence staining was performed as described elsewhere (Cordes *et al*, 2002). In brief, 5x10^3^ A-172 or U-138 cells were seeded onto Lab Tek chamber slides (Nalge Nunc International, Germany). After a 24-h incubation of cells at 37°C in a humidified atmosphere containing 10% CO_2_, cells were fixed using 2% paraformaldehyde in PBS and blocked with 2% goat serum. Staining of cells with anti-*β*1-integrin (Dako, Germany) and anti-MMP-2 (Oncogene, Germany) antibodies was performed for 1 h at room temperature at a dilution of 1 : 150 and 1 : 150, respectively. Secondary antibodies, Cy™2-conjugated AffiniPure goat anti-mouse IgG and Cy™3-conjugated AffiniPure goat anti-rat IgG were used at a dilution of 1 : 100 (Dianova, Germany). Fluorescence images were obtained using a confocal laser scan microscope equipped with TCS NT software (LEICA Instruments, Germany).

### Gelatine assay

Conditioned, serum-free media from nonirradiated or irradiated (6 Gy) cells were collected 48 h after irradiation, centrifuged to remove corpuscular material, snap frozen in liquid nitrogen and stored at –134°C until use. In parallel, cells were counted to enable analysis of MMP activity per cell number. Dependence of MMP activities on *β*1- and *β*3-integrins was also examined in cells incubated with 10 *μ*g/ml^−1^ anti-*β*1-integrin antibody mAb13, 10 *μ*g/ml^−1^ anti-*β*3-integrin antibody RUU-PL7F12 or 10 *μ*M echistatin without or in combination with 25 *μ*M MMP-2/-9 inhibitor III for 15 min in serum-free medium prior to irradiation (6 Gy). Unspecific IgG2a and IgG1 antibodies were employed for control experiments using identical concentrations as done for specific antibodies. Media equal to 1.1 × 10^4^ cells were electrophoresed on 10% SDS-gels copolymerised with 0.1% gelatine. Subsequently, gels were washed twice in 50 mM Tris-HCl (pH 7.5)/2.5% Triton X-100 for 30 min, incubated overnight at 37°C with 50 mM Tris-HCl (pH 7.6), 10 mM CaCl_2_, 150 mM NaCl and 0.05% NaN_3_, stained with Coomassie blue and destained to identify the zones of lysis.

### Data analysis

Means plus/minus standard deviations of surviving fractions and flow cytometric analysis of *β*1- and *β*3-integrin expression were calculated with reference to untreated controls defined as 1.0. To test statistical significance, analysis of variance was performed by means of ANOVA with a software package (Microsoft, Excel 97) on IBM computer systems. Results were considered statistically significant or highly significant if *P*-value of less than 0.05 or 0.01 was reached, respectively. The fit of the dose – effect curves was calculated using the linear–quadratic model (log *S*=−α*D*−*βD*^2^).

## RESULTS

### ECM presence differentially affected cell survival after irradiation

Clonogenic survival was determined to measure substratum-dependent cell survival after irradiation.

**Figure 1:**
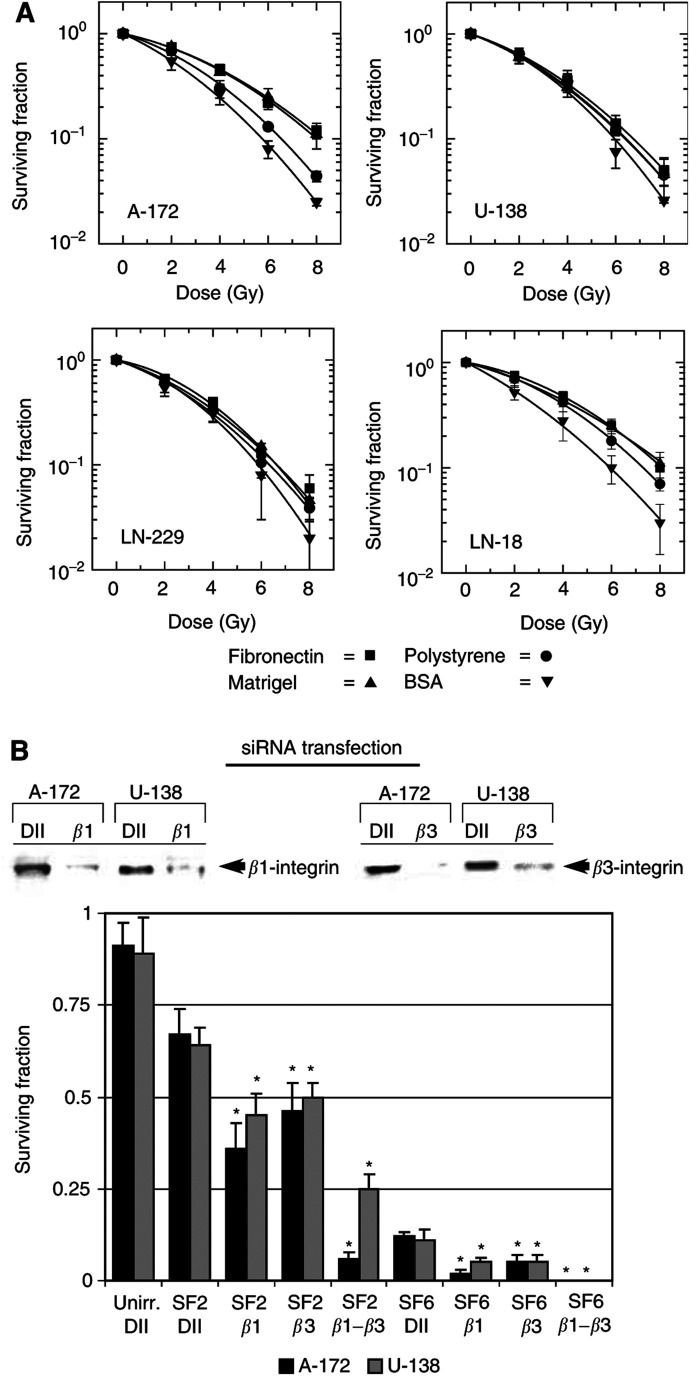
(**A**) Plating human A-172, U-138, LN-229 or LN-18 glioblastoma cells on FN, Matrigel, polystyrene or BSA differentially altered the cellular radiosensitivity. Survival of A-172 cells grown on FN or Matrigel was significantly (*P*<0.01) improved at doses ⩾4 Gy. In contrast, irradiated U-138, LN-229 or LN-18 cells showed no substratum-dependent alteration of cell survival. Each data point represents the mean ±s.d. of three independent experiments (*n*=18). (**B**) The surviving fractions after 2 (SF2) or 6 Gy (SF6) of A-172 and U-138 cells were significantly (*P*<0.01) reduced after single or combined *β*1- and *β*3-integrin depletion using siRNA transfection (see Western blot) as compared to irradiated cells transfected with unspecific Duplex II RNA (DII). Single Duplex II RNA (Unirr. DII) did not significantly reduce the plating efficiency of A-127 and U-138 cells. ^*^ *P*<0.01.

The survival of A-172 cells was significantly (*P*<0.01) improved following radiation doses ≥4 Gy when grown on FN or Matrigel (Figure 1A

). In contrast, irradiation of U-138, LN-229 and LN-18 cells adhered to FN or Matrigel resulted in surviving fractions similar to cells grown on polystyrene (Figure 1A). Attachment of cells to BSA reduced survival most effectively after irradiation in all four cell lines tested.

Silencing of *β*1- or *β*3-integrin expression by siRNA transfection of A-172 and U-138 cells resulted in significantly (*P*<0.01) reduced surviving fractions after 2 (SF2) or 6 Gy (SF6) compared to the SF2 and SF6 of untransfected cells as plotted in Figure 1A (Figure 1B). While the SF2 of A-172 and U-138 cells was dramatically decreased after double depletion of *β*1- and *β*3-integrin expression, the SF6 could not be determined due to complete cell killing (Figure 1B). Control siRNA transfections with unspecific Duplex II showed no significant cytotoxicity compared to untransfected cells (Figure 1B).

### Irradiation induced *β*1- and *β*3-integrin cell surface expression

To confirm previous findings that show radiation-induced integrin expression (Cordes *et al*, 2002, 2003), the glioblastoma cell lines were irradiated and the cell surface expression of *β*1- and *β*3-integrins was measured by FACS.

**Figure 2:**
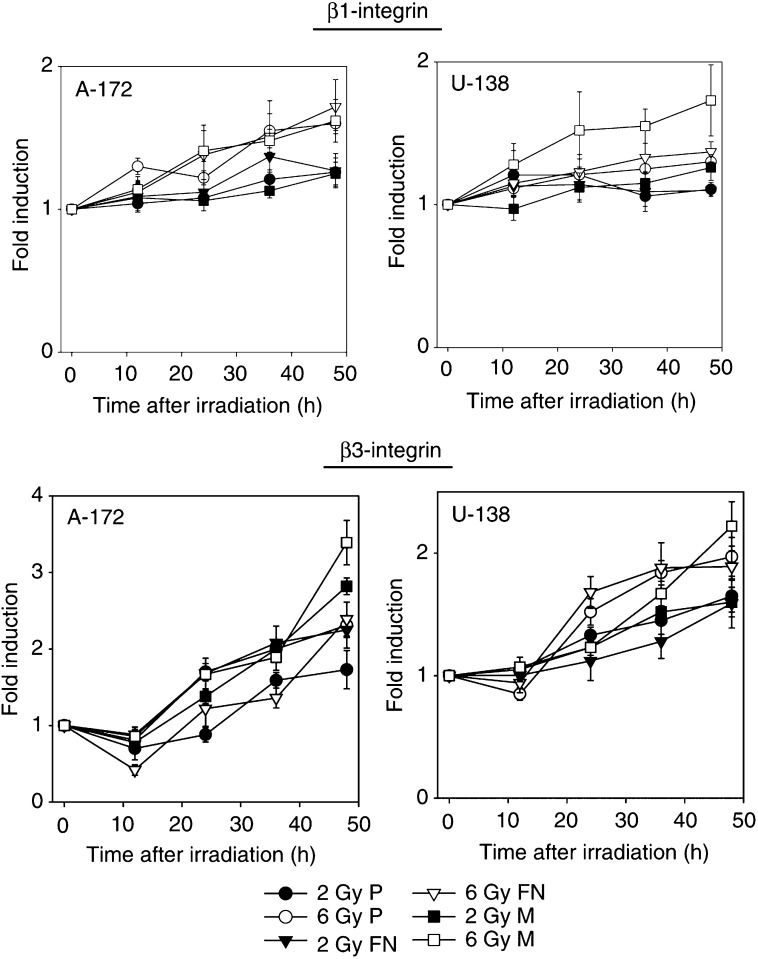
48-h flow cytometric analysis of *β*1- and *β*3-integrin cell surface expression in nonirradiated and irradiated A-172 and U-138 cells showed a dose-dependent induction of these two integrin receptors. Each data point represents the fold induction (mean ±s.d. of three independent experiments) of irradiated samples in comparison with unirradiated controls. P, polystyrene; FN, fibronectin; M, Matrigel.

Irradiation induced a significant and dose-dependent increase in *β*1- and *β*3-integrin cell surface expression in A-172 and U-138 cells (Figure 2

).

At 48 h after 2 Gy, *β*1-integrins of A-172 or U-138 cells were increased by 1.3-/1.26-/1.26-fold or by 1.1-/1.1-/1.26-fold (FN/Matrigel/polystyrene), respectively, compared to unirradiated cells. At 48 h after 6 Gy, *β*1-integrins of A-172 or U-138 cells were significantly (*P*<0.01) increased by 1.72-/1.6-/1.6-fold or by 1.3-(not significant)/1.37-(*P*<0.05)/1.73-fold (FN/Matrigel/polystyrene), respectively, compared to unirradiated cells.

At 48 h after 2 Gy, *β*3-integrins of A-172 or U-138 cells were significantly (*P*<0.01) elevated by 1.73-/2.25-/2.82-fold or by 1.65-/1.59-/1.6-fold (FN/Matrigel/polystyrene), respectively, compared to unirradiated cells. At 48 h after 6 Gy, *β*3-integrins of A-172 or U-138 cells were also significantly (*P*<0.01) elevated by 2.31-/2.39-/3.39-fold or by 1.92-/1.89-/2.22-fold (FN/Matrigel/polystyrene), respectively, compared to unirradiated cells.

According to the experimental conditions used in the *in vitro* invasion assay and the gelatine zymography, *β*1- and *β*3-integrin expression of A-172 and U-138 cells was also examined under serum-free conditions. The results obtained were not significantly different from those results generated in nonirradiated or irradiated cells grown with serum (data not shown).

### Adhesion to FN and Matrigel was significantly improved after irradiation

Adhesion experiments were performed to prove a normal adhesive function of basal and radiation-induced integrins. Additionally, it is shown that the radiation-dependent increase in integrin cell surface expression improves cell adhesion.

**Figure 3:**
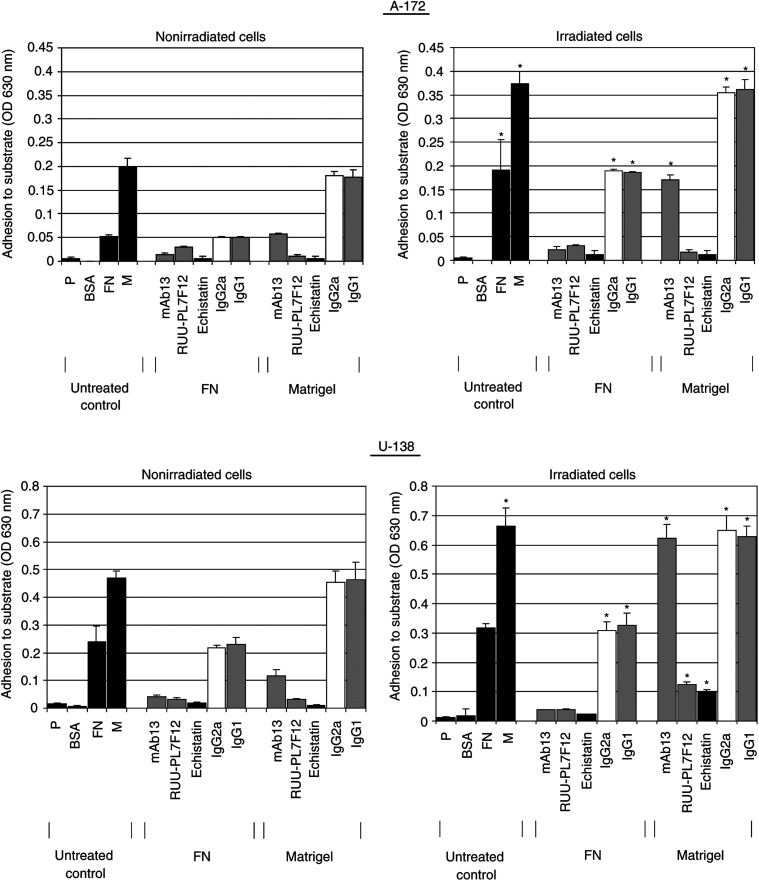
Radiation (6 Gy) improved the capability of A-172 and U-138 cells to attach to fibronectin (FN) and Matrigel after 48 h in comparison with BSA or polystyrene (P). To determine functional *β*1- and *β*3-integrin engagement in radiation-altered adhesion in these two cell lines, the cells were incubated with specific adhesion-blocking antibodies against *β*1- (mAb13) or *β*3-integrins (RUU-PL7F12) or with the disintegrin echistatin. Control experiments were performed using equivalent concentrations of unspecific anti-mouse IgG2a or IgG1 antibodies. Columns represent the mean ±s.d. of the optical densities (OD) at 630 nm representing cell adhesion to the different substrates of three independent experiments. Statistical analysis compared adhesion to FN or Matrigel of nonirradiated cells with adhesion to FN or Matrigel of irradiated cells. ^*^ *P*<0.01.

Attachment of nonirradiated A-172 and U-138 cells to FN or Matrigel was several fold greater than attachment to polystyrene or BSA (Figure 3

). Blocking of *β*1-integrins with specific antibodies impaired adhesion to FN more effectively than adhesion to Matrigel. In contrast, the anti-*β*3-integrin antibodies more effectively impaired adhesion to Matrigel than adhesion to FN. The anti-*β*1/*β*3-disintegrin echistatin blocked adhesion to both FN and Matrigel (Figure 3).

At 48 h postirradiation which represented the time point of significant radiation-dependent upregulation of *β*1- and *β*3-integrins, adhesion of irradiated A-172 cells to FN or Matrigel was significantly (*P*<0.01) increased. U-138 cells only showed significantly (*P*<0.01) increased adhesion to Matrigel (Figure 3). Blocking integrins with indicated antibodies in irradiated A-172 and U-138 cells showed similar results as obtained in unirradiated cells. Control IgG antibodies did not influence adhesion to FN or Matrigel of nonirradiated or irradiated cells.

siRNA treatment of A-172 and U-138 cells against *β*1- or *β*3-integrins caused a strong impairment of adhesion to FN and Matrigel comparable to the adhesion of untransfected cells to polystyrene. Combined depletion of both integrins completely prevented adhesion of both cell lines to polystyrene, FN or Matrigel (data not shown).

### Irradiation differentially affected cell invasion into Matrigel

Invasion is one of the major pathophysiological characteristics of glioblastoma cells. To evaluate the impact of irradiation on the invasive potential and the impact of the radiation-dependent increase of *β*1- and *β*3-integrins of the cell lines examined, the Matrigel *in vitro* invasion assay was performed.

**Figure 4:**
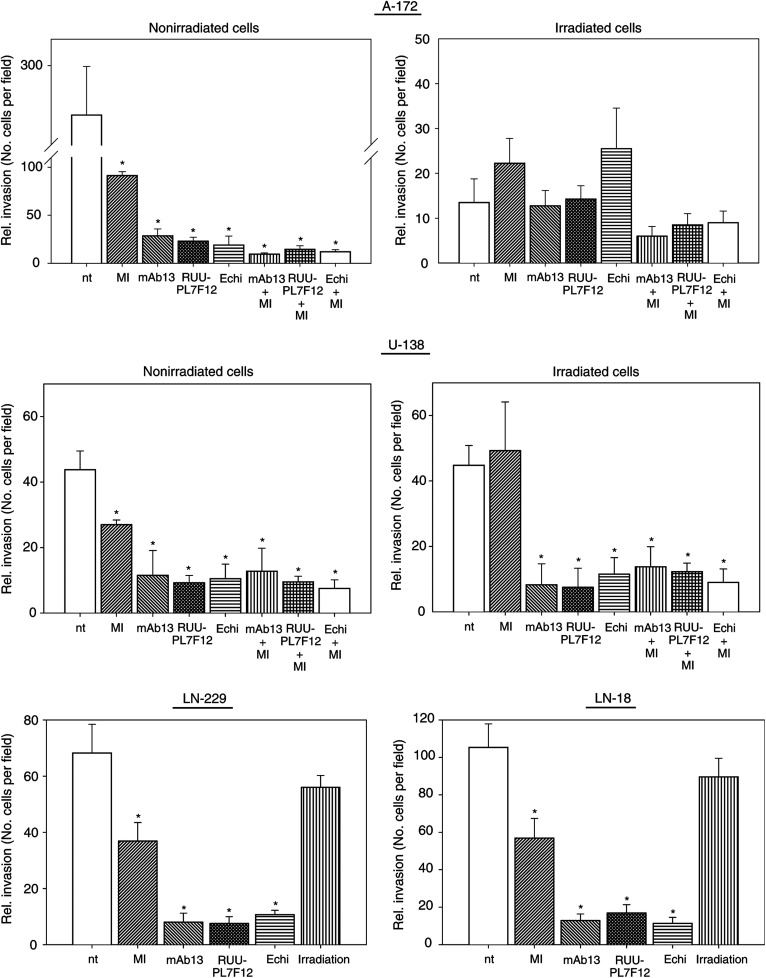
The *in-vitro* invasion assay indicated that irradiation (6 Gy) is able to inhibit strongly A-172 but not U-138, LN-229 and LN-18 cell invasion into Matrigel. To analyse the dependence of the invasion process on MMP-2 or *β*1- or *β*3-integrins, nonirradiated as well as irradiated cells were either incubated with the MMP-2/-9 inhibitor III (MI), anti-*β*1- (mAb13) and anti-*β*3-integrin (RUU-PL7F12) antibodies or the *β*1-/*β*3-integrin-blocking disintegrin echistatin alone or in combination. Additionally, control experiments were performed using equivalent concentrations of unspecific anti-mouse IgG2a or IgG1 antibodies. The data were collected by counting the number of cells per field (four high-powered fields). Columns represent the calculated mean ±s.d. of three independent experiments. Statistical significance was calculated by comparing the rate of invasion after anti-MMP and/or anti-integrin treatment to the rate of invasion of untreated cells. Furthermore, statistics were calculated by comparing the rate of invasion of treated cells after irradiation to the rate of invasion after irradiation alone. nt, no treatment; ^*^P<0.01.

The basal invasive potential of A-172 and U-138 cells into Matrigel differed by seven-fold (Figure 4

). Treating cells with the MMP-2/-9 inhibitor III reduced invasion by 3- (A-172) or 1.8-fold (U-138). Treatment of cells with anti-*β*1- or anti-*β*3-integrin antibodies or echistatin reduced invasion significantly (*P*<0.01) by 10- (A-172) or 4-fold (U-138) compared to untreated controls. Combined exposure using either MMP inhibitor plus anti-integrin antibodies or MMP inhibitor plus echistatin further decreased the degree of invasion in comparison to the single treatment conditions. Comparing LN-229 and LN-18 cells to U-138 cells, great similarity could be observed concerning their basal invasive potentials and their behaviour after treatment with MMP inhibitor and anti-integrins (Figure 4). These findings indicated a cooperation between MMP-2 and *β*1- and *β*3-integrins in the invasion of the tested cell lines (Figure 4).

Following irradiation, invasion of A-172 cells was impeded by 26-fold, whereas U-138, LN-229 and LN-18 cell invasion was not affected (Figure 4). Pretreatment of irradiated A-172 and U-138 cells with anti-integrins alone or with anti-integrins plus MMP inhibitor reduced the rate of invasion similar to the effects seen in treated, nonirradiated cells (Figure 4).

Following siRNA transfection, invasion of both A-172 and U-138 cells into Matrigel was strongly reduced by >95%. These results were independent of single or double *β*1/*β*3-integrin depletion (data not shown).

### Expression of MMP-2, MT1-MMP and TIMP-2 was affected by IR and anti-integrin treatment

Protein expression profiles of the important ECM-degrading proteinases MMP-2 and MT1-MMP and the antagonist TIMP-2 were determined to estimate changes in MMP-2 activity after radiation or treatment with anti-integrins or MMP-2/-9 inhibitor III more precisely.

**Figure 5:**
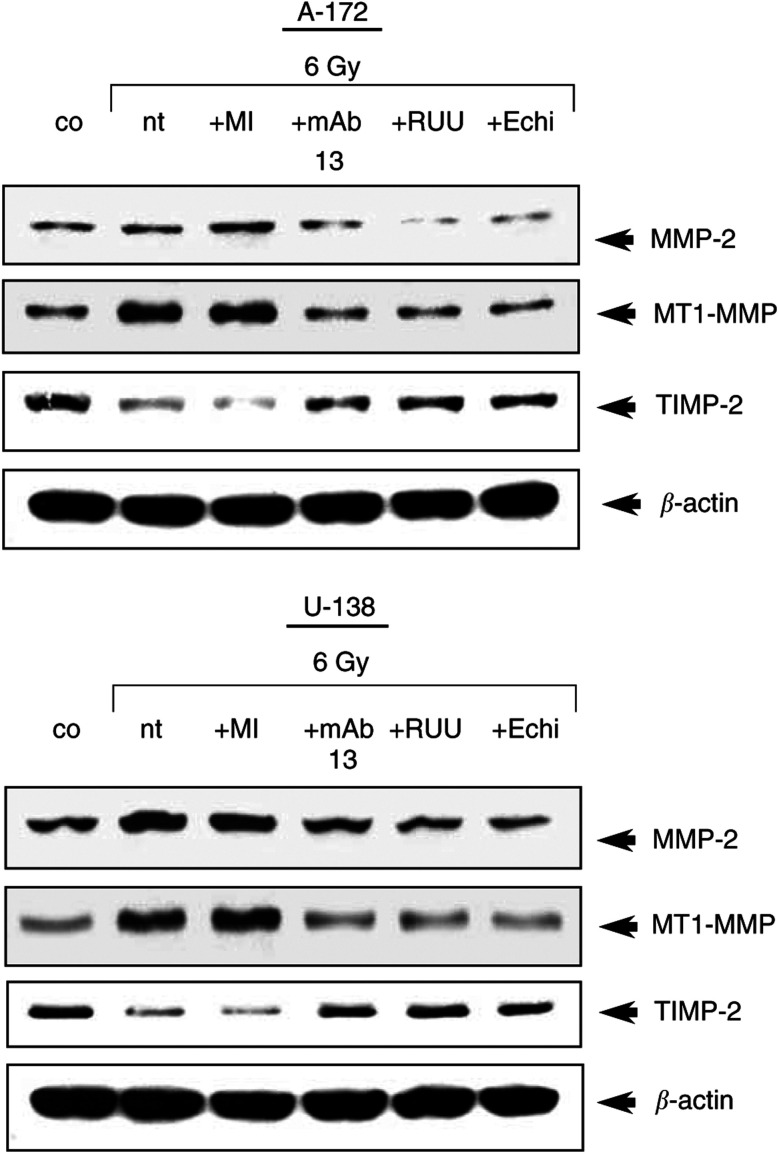
Western blot analysis of MMP-2, MT1-MMP and TIMP-2 in nonirradiated (co) and irradiated cells showed that irradiation induced MMP-2 and MMT1-MMP expression within 48 h. Treatment of cells with MMP-2/-9 inhibitor III (MI), anti-*β*1- (mAb13) or anti-β3-integrins (RUU-PL7F12, RUU) or echistatin (Echi) prevented induction of MMP-2 and MT1-MMP and promoted induction of TIMP-2 after irradiation. In contrast, TIMP-2 expression was downregulated after irradiation and induced by anti-integrin treatment. nt, no treatment.

Irradiation strongly induced MMP-2 and MT1-MMP expression in A-172 and U-138 cells (Figure 5

). While anti-integrin antibodies or echistatin prevented this radiation-dependent upregulation of MMP-2 and MT1-MMP expression, MMP-2/-9 inhibitor III had no effect. TIMP-2 expression decreased after irradiation and was stabilised by anti-integrin treatment (Figure 5).

### *β*1-integrins colocalised with MMP-2 at distinct regions of the cell membrane

To confirm reported observations about membranous *β*1-integrin-MMP-2 colocalisation at sites called focal adhesions which represent the structural and signalling correlate that connect the ECM to the cytoskeleton, confocal scanning microscopy was performed.

**Figure 6:**
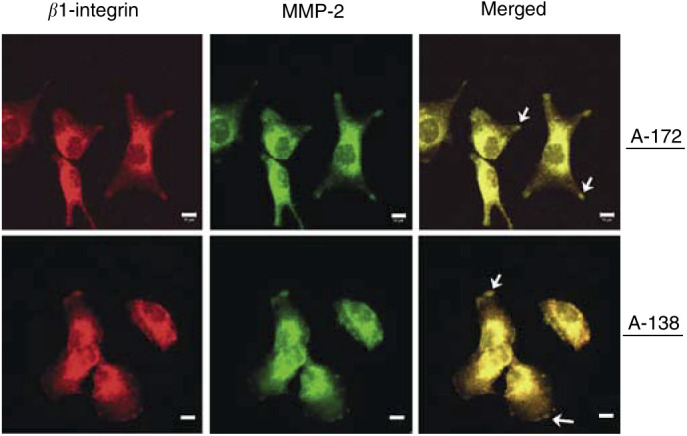
Double immunofluorescence staining of *β*1-integrin plus MMP-2 in A-172 and U-138 cells. β1-integrin and MMP-2 were visualised by confocal scanning microscopy after fixing and staining of cells with the appropriate antibodies. Arrows indicate *β*1-integrin plus MMP-2 colocalisation within focal adhesion sites in the cell membrane. Bar, 10 *μ*m.

The fluorescence signals of *β*1-integrins in combination with MMP-2 colocalised at these specific membrane regions termed focal adhesions and in the cytoplasm (Figure 6

). In control experiments using the secondary antibodies, no fluorescence signals could be detected. Similar results were obtained in LN-229 and LN-18 cells (data not shown).

### Gelatinolytic activity of MMP-2 was strongly modulated by irradiation, anti-integrins and MMP-inhibitor

The gelatinolytic activity of MMP-2, an important matrix-cleaving proteinase, was measured to define the effects of radiation and anti-integrins or MMP inhibitor treatment with regard to cell invasion in more detail.

**Figure 7:**
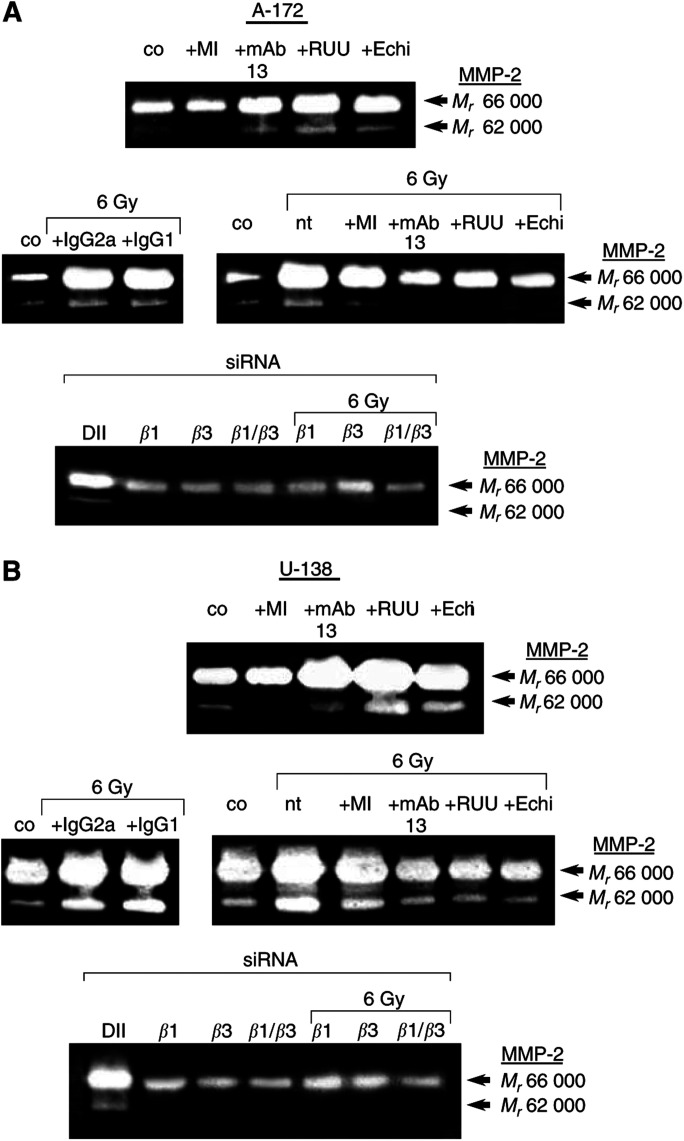
Conditioned media of 48-h nonirradiated (co) and irradiated A-172 (**A**) or U-138 (**B**) cells were analysed on 10% PAGE-gelatine. Effects of MMP-2/-9 inhibitor III (MI), anti-*β*1-integrin (mAb13) and anti-*β*3-integrin (RUU-PL7F12, RUU) antibodies and the *β*1-/*β*3-blocking disintegrin echistatin on the gelatinolytic activity of MMP-2 and its conversion from the pro (*M*_*r*_ 66 000) to the active form (*M*_*r*_ 62 000) are shown in nonirradiated and irradiated cells. Additionally, irradiation control experiments were performed using equivalent concentrations of unspecific anti-mouse IgG2a or IgG1 antibodies. Following *β*1- and/or *β*3-integrin siRNA transfection of A-172 and U-138 cells, the gelatinolytic activity of MMP-2 was strongly decreased in both cell lines. The combined depletion of both *β*1- and *β*3-integrin had the greatest effect. nt, no treatment; DII, unspecific Duplex II control.

Basal MMP-2 activity was reduced by MMP-2/-9 inhibitor III in both cell lines (Figure 7A and B

). Treatment of cells with anti-integrins showed an induction of pro-MMP-2 (*M*_*r*_ 66 000) and an increased conversion of pro-MMP-2 to the active form (*M*_*r*_ 62 000) (Figure 7A and B). These findings were likely to be due a stimulation of integrins and their signalling pathways by the employed antibodies.

Irradiated A-172 (Figure 7A) and U-138 (Figure 7B) cells showed increased gelatinolytic activity of MMP-2 within 48 h. While the MMP inhibitor slightly reduced the conversion of MMP-2 to its active form in irradiated A-172, it demonstrated to be less effective in irradiated U-138 cells. The radiation-induced MMP-2 activity and the enhancement of MMP-2 conversion was prevented by anti-*β*1- and anti-*β*3-integrin antibodies or echistatin in both cell lines. Treatment of cells with the combination of anti-integrin antibodies plus MMP-2/-9 inhibitor III resulted in similar data as shown for nonirradiated or irradiated controls (data not shown). Exposure of A-172 (Figure 7A) and U-138 cells (Figure 7B) with unspecific IgG2a and IgG1 control antibodies did not prevent radiation-dependent induction of MMP-2 activity as observed in untreated, irradiated cells.

Depleting *β*1- or *β*3-integrins by siRNA transfection of A-172 and U-138 cells strongly decreased MMP-2 activity. A combined depletion of *β*1- plus *β*3-integrins presented most effective in reducing the gelatinolytic activity of MMP-2 (Figure 7A and B).

## DISCUSSION

Effects of irradiation on ECM-modulated cell survival as well as on adhesion and invasion are not well understood. Major findings of this study include: (i) a significantly reduced radiosensitivity of A-172 but not of U-138, LN-229 and LN-18 cells grown on FN or Matrigel compared to cells grown on polystyrene or BSA; (ii) a significantly induced upregulation of functional *β*1- and *β*3-integrins by irradiation that could be correlated with improved *β*1- and *β*3-integrin-mediated adhesion to FN or Matrigel; (iii) strong invasion-inhibiting effects of irradiation on A-172 cells in contrast to U-138, LN-229, and LN-18 cells; (iv) a modulating role of *β*1- and *β*3-integrins on the gelatinolytic activity of MMP-2 without and in combination with irradiation; and (v) siRNA transfection data that strongly underlined *β*1- and *β*3-integrins as key molecules in glioma cell survival, adhesion, and invasion.

The examination of various transformed cell lines and primary cell strains has raised evidence for chemo- and radioprotective effects of specific ECM proteins (Vlodavsky *et al*, 1980; Fuks *et al*, 1992; Rose *et al*, 1999; Hazlehurst *et al*, 2001; Cordes *et al*, 2002 in press (a), 2003; Cordes and Meineke, 2003; Cordes and van Beuningen, 2003). As reported (Bouterfa *et al*, 1999; Oz *et al*, 2000; Mahesparan *et al*, 2003), FN, laminin, collagen-IV and -I, decorin, tenascin and vitronectin are ECM proteins produced and secreted by cells of malignant tumours of glial origin *in vitro* and *in vivo*. Although it is difficult to correlate heterogeneous ECM protein expression patterns in the same or among different tumours and migratory and invasive behaviour, the findings suggest that glioma cells are able to modulate their microenvironment thus affecting survival, migration and invasion. Clonogenic survival data generated in the glioma cell line A-172 grown on FN or the basement membrane matrix equivalent, Matrigel, confirmed that certain glioma cell lines are affected by matrix proteins. This results in an adhesion-mediated radioprotective effect *in vitro*. The cellular susceptibility to matrix proteins seems to be a prerequisite for the modulation of the intrinsic cellular radiosensitivity. For adhesion to artificial and physiological surfaces, adherent growing cells recruit cell adhesion molecules. However, in case of adhesion to a physiological ECM protein many, if not all, cell functions are specifically modified by integrin-mediated signalling (Albelda, 1993; Assoian, 1997; Giancotti and Ruoslahti, 1999; Schwartz and Assoian, 2001). In particular, survival-improving signalling has been tightly connected with the widespread integrin subunit *β*1 (Meredith *et al*, 1993; Schwartz, 2001). In contrast to A-172 cells, irradiated U-138, LN-229, and LN-18 cells that were unsusceptible to the used substrates showed ECM-independent survival. Specific genomic mutations or post-transcriptional modifications of molecules that are substantially involved in cell-matrix-stimulated and survival-advantaging signalling have to be taken into consideration. Interestingly, the clonogenic survival data of *β*1- and *β*3-integrin depleted A-172 and U-138 cells questions this hypothesis, because both cell lines demonstrated a great increase in cell killing after irradiation in comparison with untransfected, irradiated or control Duplex II-transfected, irradiated cells. With regard to findings of Lewis *et al* (2002), who observed adhesion-mediated apoptosis in certain transformed cell lines after DNA damage, the killing of these cell lines could be increased by treatment with anti-integrin antibodies and increased levels of p53 or Arf. The cell lines used in our study displayed either wild-type p53 (A-172) or mutant p53 (U-138, LN-229, LN-18). Preliminary data from our lab show that basal p53 expression levels and levels of downstream targets such as p21 are not altered by the substratum itself on which cells are growing on (Cordes, 2003, unpublished observation). FN adhered p53-wild-type cell cultures, for example, normal human fibroblasts or A549 lung cancer cells, demonstrate delayed changes of p53 and p21 in response to DNA damage compared to cell cultures adhered to polystyrene. In contrast to protein expression, the induction of p53 phosphorylation on serine-20 is accelerated in FN cultures after irradiation compared to polystyrene cultures (Cordes and Beinke, in press). Additionally, human p53-wild-type fibroblasts or A-172 glioma cells accumulate more strongly in the G2-phase of the cell cycle after irradiation and show an improved ECM-dependent radiation survival response. Further investigations will elucidate whether mutated p53 or Arf are responsible for the different glioma cell behaviours observed in this study.

Integrins serve as adhesion receptors to matrix proteins. Irradiation improved adhesion of A-172 and U-138 cells to FN or Matrigel through an upregulation of functional *β*1- and *β*3-integrins on the cell surface. Blocking integrins with specific antibodies or echistatin revealed that attachment of these cells to FN is mainly mediated by *β*1-integrins, while attachment to the basement membrane matrix equivalent, Matrigel, is mainly enabled by *β*3-integrins. As these effects were observed in both nonirradiated and irradiated cells, the integrin recruitment during adhesion seems to be matrix-specific and not to be influenced by irradiation. Depletion of *β*1- and *β*3-integrins in A-172 and U-138 cells strongly supported these findings and almost completely abrogated the cell's adhesive capability.

Adhesion and migration are closely linked cell functions. Thus, changes in integrin patterns are likely to affect both of these processes. MMP-2, as integral molecule of the invasion process, was activated by irradiation in a *β*1- and *β*3-integrin-dependent manner and by stimulating anti-*β*1- and anti-*β*3-integrin antibodies. In controversy with the well-known association of the invasive phenotype with MMP overexpression (Rich *et al*, 1985; Ballin *et al*, 1988), the radiation-mediated MMP-2 induction (Engebraaten *et al*, 1992; Nirmala *et al*, 2000; Wild-Bode *et al*, 2001) did not lead to increased A-172 and U-138 invasion. Our controversial data suggest, on the one hand, cell line-specific interactions between basal gelatinolytic MMP-2 activity and the degree of invasiveness and, on the other hand, an inverse cell line-specific interaction between radiation-induced MMP-2 activity and radiation-dependent impairment of invasion. MMP-2 has been reported to colocalise with *α*v*β*3- (Felding-Habermann and Cheresh, 1993; Brooks *et al*, 1996) and with *β*1-integrin (Ellerbroek *et al*, 1999). Confocal scanning microscopy of A-172 and U-138 cells confirmed the colocalisation of *β*1-integrins and MMP-2 at focal adhesion sites that are the morphological correlates responsible for structural and regulatory cell–matrix interactions within, for example adhesion or invasion. Furthermore, the close proximity of MMPs and integrins at these sites enables direct coordination of migration and invasion processes. The findings presented here could be based on a radiation-dependent modification of *β*1/*β*3-MMP-2 interactions as well as intermolecular affinities. Our data suggest that *β*1- and *β*3-integrin aggregation and occupancy by specific antibodies block important cell motility-related receptor epitopes. These events result in a local anchorage of treated cells. This anchorage is likely to cause a reduction of MMP-2-specific, membrane-located substrates. We hypothesise that the radiation-mediated upregulation of MMP-2-stimulating *β*1- and *β*3-integrins in the combination with the radiation-induced MMP-2 activity promotes a reduction of MMP-2 substrates. Consequently, the cells are not able to migrate and invade accordingly. In contrast to A-172 cells which fit into this model, U-138, LN-229 and LN-18 cells displayed a much higher basal gelatinolytic MMP-2 activity and lower invasive potential. Treatment of U-138 cells with the MMP inhibitor completely failed to inhibit invasion. The phenotype of U-138, LN-229 and LN-18 cells does therefore rather fit into another model. As already reported, a high MMP activity is not consequently bound to a high invasive potential because an extensive uncontrolled ECM-degradation can impede cell invasion due to a lack of required matrix components (Ruoslahti, 1992; Montgomery *et al*, 1994). Invasion of U-138, LN-229 and LN-18 cells seems to substantially depend on integrin function rather than on different MMP-2 levels. Further evidence for a direct cooperation between *β*1- and *β*3-integrins and MMP-2 was raised by silencing of both of these integrins in A-172 and U-138 cells. The single and combined depletion led to a strong decrease of the gelatinolytic activity of pro-MMP-2 and a complete loss of its active form.

Examination of MMP-2, MT1-MMP, a membrane-bound MMP which is thought to facilitate rapid processing of pro-MMP-2 (Chintala *et al*, 1999), and TIMP-2 showed that the expression patterns of MMP-2 and MT1-MMP were very similar among the examined glioma cell lines. MMP-2 and MT1-MMP were induced in irradiated cell cultures and they were reduced in irradiated cell cultures pretreated with anti-integrins. TIMP-2 which preferentially inhibits MMP-2 (Corcoran *et al*, 1996) showed to be expressed inversly compared to MMP-2 and MT1-MMP. In LN-229 and LN-18 cells TIMP-2 was not detectable.

In summary, the presented findings indicate differential radiosensitivity-modulating effects of FN and the basement membrane equivalent matrix mixture, Matrigel, on the glioma cell lines A-172, U-138, LN-229 and LN-18. Three out of four tested glioma cell lines demonstrated a combination of a substratum-independent radiation survival response and an invasive potential which was not affected by irradiation. Furthermore, we can provide a mechanistic link between *β*1- or *β*3-integrins and MMP-2 and may have implications for models of tumour cell invasion. The role of radiation-mediated induction of *β*1- and *β*3-integrins remains elusive in detail, but may partly account for altered glioma cell invasion. Finally, our data might offer a new therapeutic strategy, in which ligating integrins or inhibiting relevant integrin pathways may be effective as an adjunct to standard chemo- and radiotherapy to enforce cell killing and impair cell invasion of lethal high-grade astrocytoma.
